# Enhanced Charge Carrier Separation in WO_3_/BiVO_4_ Photoanodes Achieved via Light Absorption in the BiVO_4_ Layer

**DOI:** 10.1021/acsaem.2c02597

**Published:** 2022-10-17

**Authors:** Ivan Grigioni, Annalisa Polo, Maria Vittoria Dozzi, Kevin G. Stamplecoskie, Danilo H. Jara, Prashant V. Kamat, Elena Selli

**Affiliations:** † Dipartimento di Chimica, Università degli Studi di Milano, Via Golgi 19, Milano20133, Italy; ‡ Department of Chemistry, Queen’s University, Kingston, OntarioK7L 3N6, Canada; § Facultad de Ingeniería y Ciencias, Universidad Adolfo Ibáñez, Avenida Padre Hurtado 750, Viña del Mar7941169, Chile; ⊥ Radiation Laboratory, University of Notre Dame, Notre Dame, Indiana46556, United States

**Keywords:** solar water oxidation, heterojunction, ultrafast transient absorption, photoactive layer thickness, filter effect

## Abstract

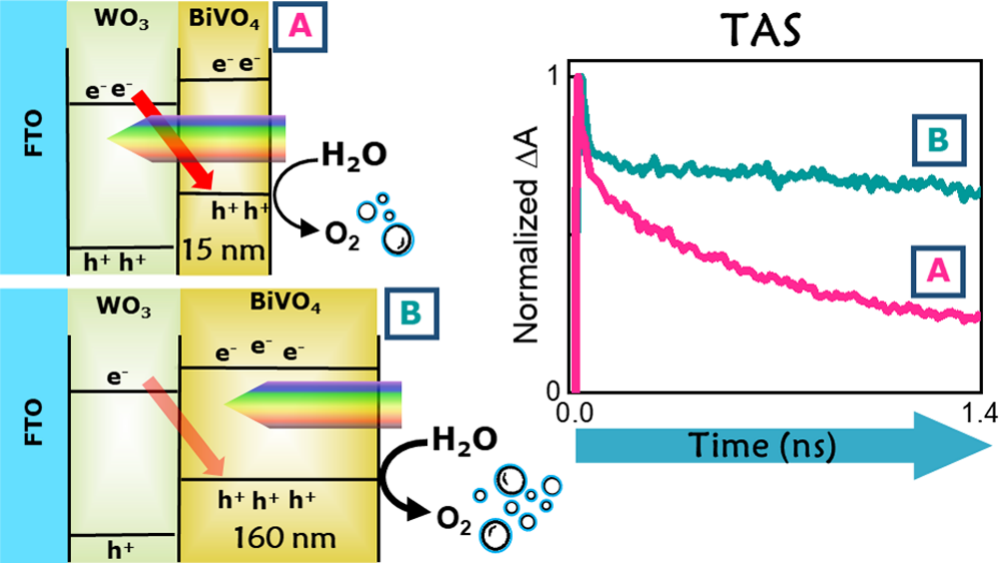

Photoelectrochemical (PEC) water splitting converts solar light and water into oxygen and energy-rich hydrogen. WO_3_/BiVO_4_ heterojunction photoanodes perform much better than the separate oxide components, though internal charge recombination undermines their PEC performance when both oxides absorb light. Here we exploit the BiVO_4_ layer to sensitize WO_3_ to visible light and shield it from direct photoexcitation to overcome this efficiency loss. PEC experiments and ultrafast transient absorption spectroscopy performed by frontside (through BiVO_4_) or backside (through WO_3_) irradiating photoanodes with different BiVO_4_ layer thickness demonstrate that irradiation through BiVO_4_ is beneficial for charge separation. Optimized electrodes irradiated through BiVO_4_ show 40% higher photocurrent density compared to backside irradiation.

Bismuth vanadate, BiVO_4_, is a promising semiconductor oxide employed in photoanodes for the oxygen evolution reaction in water-splitting devices.^1,2^ Its stability in contact with aqueous electrolytes,^3,4^ its good visible light-harvesting capability,^5^ and its simple preparation through cheap wet techniques^6^ point to this material as a possible component of future commercial photoelectrochemical (PEC) cells. Furthermore, in the last 15 years the efficiency of BiVO_4_-based photoanodes (in terms of current density) rapidly grew from a few microamps per square centimeter in early reports to 4–5 mA cm^–2^, with prolonged continuous operation of photoelectrodes modified with oxygen evolution cocatalysts.^3,4,6−13^ However, the fast charge recombination of BiVO_4_-based electrodes still hampers the efficiency of this material.^2,14^

A way to overcome this intrinsic flaw is to couple BiVO_4_ with WO_3_ in the WO_3_/BiVO_4_ heterojunction where visible light harvesting BiVO_4_ sensitizes wider band gap WO_3_.^15^ BiVO_4_ photoanodes based on this heterojunction achieve the highest current densities among oxide-based photoanodes.^16,17^ The suitable band gap alignment between the two oxides, the efficient electron and hole transport in WO_3_ and BiVO_4_, respectively, and the spacial charge separation support the high performance of WO_3_/BiVO_4_ photoanodes.^18−25^

In previous studies, we investigated the charge carrier dynamics in the WO_3_/BiVO_4_ system through transient absorption spectroscopy (TAS) with detection either in the visible range to observe the hole dynamics in BiVO_4_^18,26−28^ or in the mid-infrared to follow the electron dynamics in WO_3_ and BiVO_4_.^29^ We also identified wavelength-dependent processes by tuning the excitation wavelength across the WO_3_ absorption edge (ca. 450 nm).^18,26,29^ Indeed, the type II band alignment between the two oxides (Figure 1A) allows distinct charge transfer processes leading to charge separation or recombination, depending on the excitation wavelength. Under visible light excitation of BiVO_4_, electrons promoted in its conduction band (CB) flow into the energetically lower-lying CB of WO_3_, while holes remain in the BiVO_4_ valence band (VB). This electron transfer process (process Ⓐ in Figure 1A) decreases charge recombination and leads to long-living charge carriers that are beneficial for PEC performance.^29^

**Figure 1:**
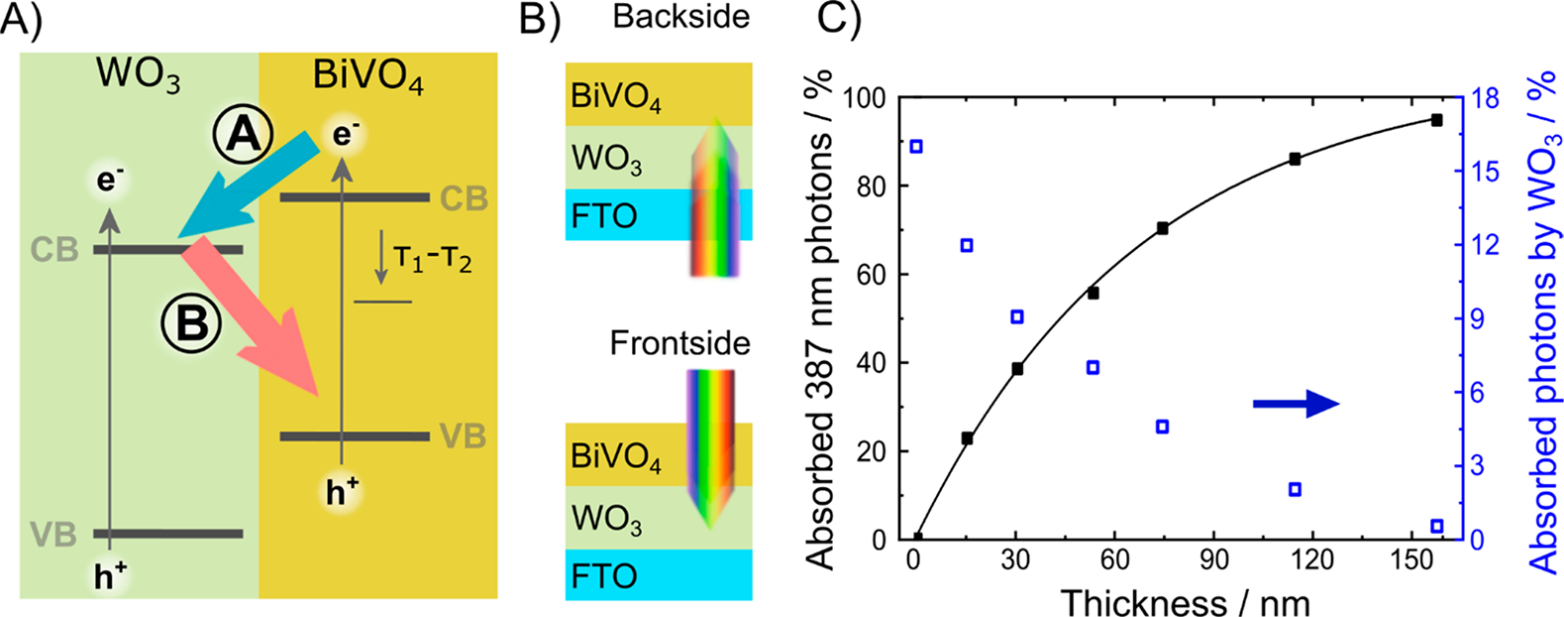
(A) Charge carrier transfer paths involved in the WO_3_/BiVO_4_ heterojunction: light absorption in BiVO_4_ produces valence band (VB) holes h^+^ and conduction band (CB) electrons e^–^, which can recombine with holes (with time constants τ_1_ and τ_2_) or flow into WO_3_ through process Ⓐ, leading to charge separation. When WO_3_ is also photoexcited, electrons in its CB can recombine with holes in BiVO_4_ through process Ⓑ. (B) Backside (through WO_3_) and frontside (through BiVO_4_) irradiation modes of the WO_3_/BiVO_4_ heterojunction photoanodes. (C) Percent amount of 387 nm photons (the pump wavelength used in TAS experiments) absorbed by the BiVO_4_ layer (left ordinate) and by the WO_3_ layer (right ordinate) under frontside excitation vs the thickness of the BiVO_4_ layer in WO_3_/BiVO_4_ photoanodes.

Conversely, irradiation at wavelengths below 450 nm leads to the excitation of both oxides and opens a detrimental recombination path between the electrons photopromoted in the CB of WO_3_ and the holes in BiVO_4_ (process Ⓑ in Figure 1A). This process results in charge recombination on a ∼200 ps time scale^26^ and becomes more relevant with increasing WO_3_ layer thickness.^30^

Based on these dynamics, we posited that an efficient heterojunction system needs to direct charges along process Ⓐ and disfavor off-track routes such as process Ⓑ. Still, solar light includes photons energetic enough to excite WO_3_ (∼4% of the solar spectrum is at wavelengths below the absorption edge of bulk WO_3_). Therefore, a portion of photogenerated charges in WO_3_/BiVO_4_ systems may be wasted through process Ⓑ. On the other hand, BiVO_4_ efficiently absorbs light beyond the absorption edge of WO_3_, up to 520 nm, allowing us to exploit a larger fraction of the solar spectrum. Therefore, in this work we pursue the strategy of using the BiVO_4_ sensitizer to shield WO_3_ from direct photoexcitation.

We assembled a series of heterojunction electrodes with a WO_3_ scaffold layer of fixed thickness (ca. 150 nm) coated with BiVO_4_ overlayers with different thickness (15–160 nm) to tune the amount of light absorbed by BiVO_4_. First, a systematic PEC study allowed us to probe whether the irradiation mode (through WO_3_ or BiVO_4_, backside or frontside irradiation, respectively, Figure 1B) affects the overall PEC efficiency of the electrodes. Then, transient absorption spectroscopy (TAS) with a pump in the UV region (387 nm) and detection in the visible range was employed to assess the effects of the irradiation mode on the lifetime of photogenerated holes in BiVO_4_. These tests allowed us to evaluate the extent of charge recombination induced by process Ⓑ and its impact on the PEC performance of the heterojunction photoanodes as a function of the BiVO_4_ layer thickness.

The WO_3_/BiVO_4_ photoanodes were prepared through spin coating using fluorine-doped tin oxide (FTO) as the conductive glass substrate (see the Supporting Information). The heterojunction electrode with the thickest BiVO_4_ layer almost entirely absorbs 387 nm photons, the pump wavelength in TAS experiments, Figure 1C. A series of control photoanodes consisting of pure BiVO_4_ on FTO (without WO_3_ layer) with variable BiVO_4_ thickness was also prepared. The absorption spectra of the two electrode series are shown in Figures S1 and S2; the thickness of the BiVO_4_ layer was estimated using the absorption coefficient at 420 nm,^19^ α_40_ = 6.7 × 10^4^ cm^–1^. XRD analyses confirm the successful synthesis of WO_3_ and BiVO_4_ (Figure S3) and FESEM images demonstrate the uniform coating of the photoanodes (Figure S4).

In order to explore the shielding hypothesis, we carried out PEC experiments on the electrodes. Figure 2A, B shows the photocurrent density generated with the WO_3_/BiVO_4_ electrodes under simulated solar light irradiation in 0.5 M Na_2_SO_4_ solution under back- and frontside irradiation at different applied potentials. The linear sweep voltammetry plots are reported in Figures S5 and S6. As a general trend, all heterojunction photoanodes outperform control pure BiVO_4_ electrodes (see Figure S7). Furthermore, the better light exploitation achieved with increasing the BiVO_4_ layer thickness drives the photocurrent increase under both irradiation conditions up to a 75 nm thick BiVO_4_ layer.

**Figure 2:**
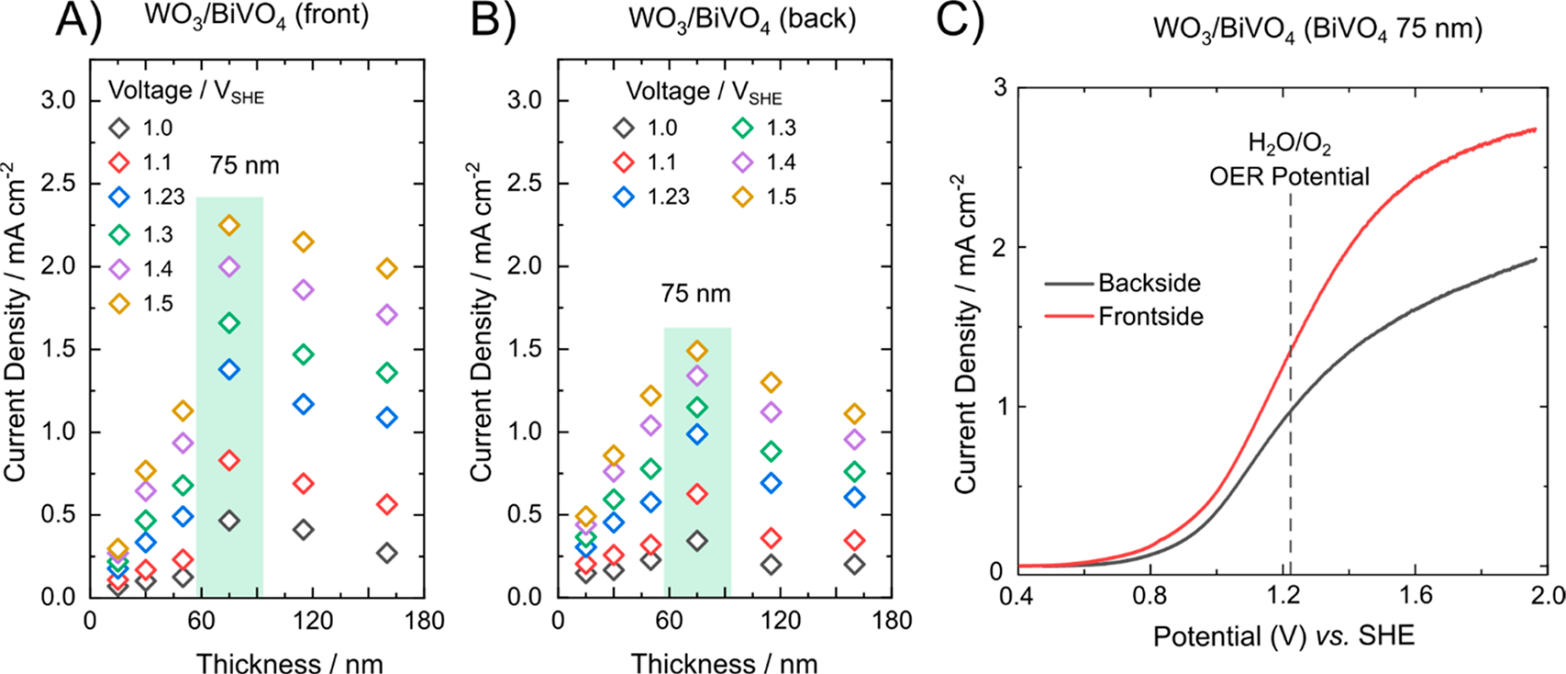
Current density generated under 1 sun simulated solar light (AM 1.5 G irradiation) at different applied potentials with the WO_3_/BiVO_4_ heterojunction electrodes in contact with a 0.5 M Na_2_SO_4_ aqueous solution under (A) frontside and (B) backside irradiation; (C) linear sweep voltammetry recorded with the best performing WO_3_/BiVO_4_ electrode (with a 75 nm thick BiVO_4_ layer) under backside (black line) and frontside (red line) irradiation; the vertical dashed line indicates the standard oxygen evolution reaction potential.

Under frontside simulated solar light irradiation (Figure 2A), the heterojunction photoanodes generate considerably higher photocurrent than in backside mode (Figure 2B). The best performing electrode with a 75 nm BiVO_4_ layer thickness (Figure 2C), when irradiated frontside shows a ca. 40% increase in the current density, from 1.0 to 1.38 mA cm^–2^, with respect to backside irradiation, at the formal H_2_O/O_2_ redox potential of 1.23 V vs the standard hydrogen electrode (V_SHE_).

We used single-wavelength efficiency measurements to gather further information on this PEC performance increase. Specifically, internal quantum efficiency (IQE, Figure 3 and Figures S8 and S9), measuring the efficiency of absorbed photons, was calculated from the incident photon to current efficiency (IPCE, see Figures S10 and S11) recorded with the WO_3_/BiVO_4_ electrodes in contact with a 0.5 M Na_2_SO_4_ solution at 1.23 V_SHE_. Figure 3A, B shows the IQE vs BiVO_4_ thickness contour plots measured under frontside and backside irradiation.

**Figure 3:**
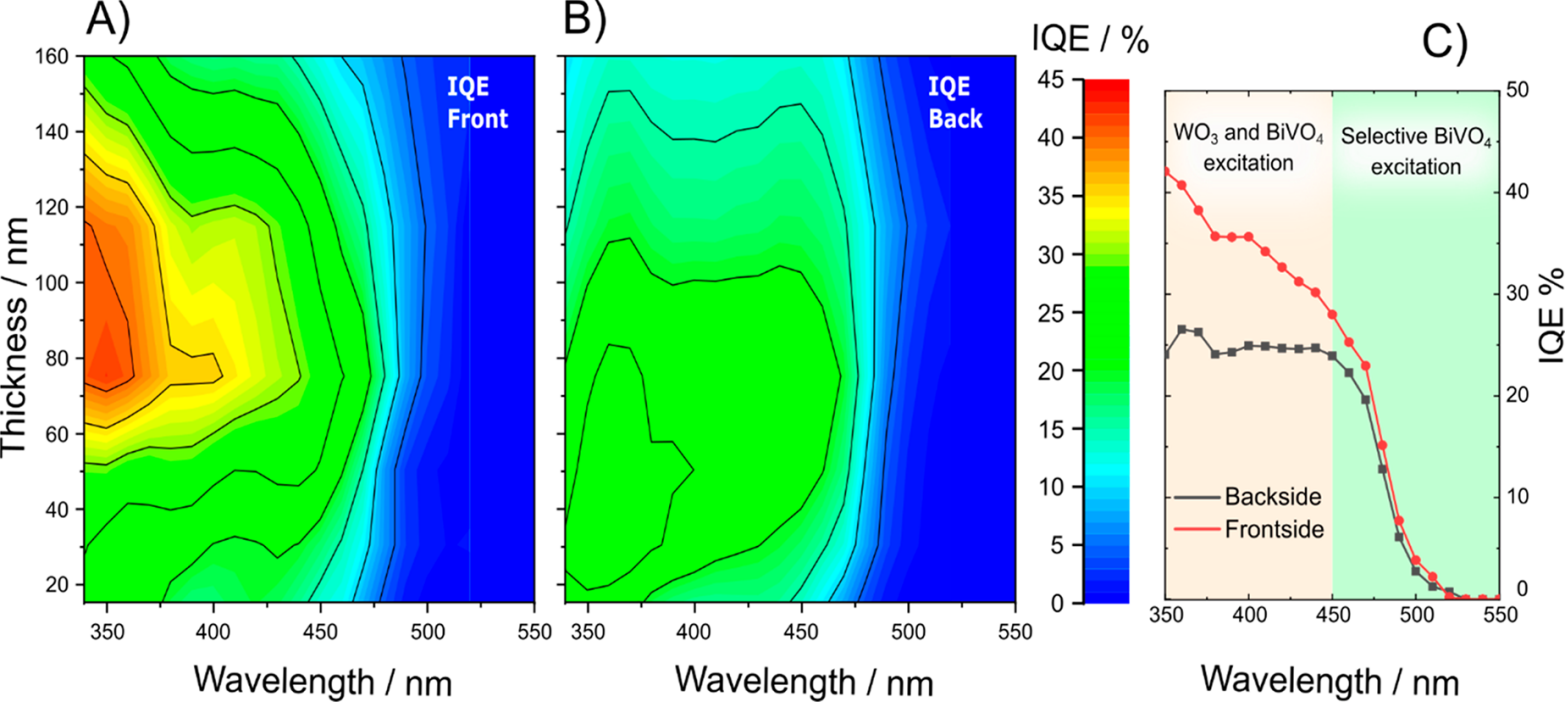
Internal quantum efficiency (IQE) 3D contour plots (color scale) vs the incident wavelength and the BiVO_4_ film thickness of the WO_3_/BiVO_4_ electrodes, obtained in contact with a 0.5 M Na_2_SO_4_ aqueous solution at 1.23 V_SHE_ under (A) frontside or (B) backside irradiation; (C) IQE plots under backside and frontside irradiation obtained with the best performing electrode (with a 75 nm thick BiVO_4_ layer).

Under frontside irradiation, the IQE reaches the highest values for 70–130 nm thick BiVO_4_ layers in the WO_3_/BiVO_4_ heterojunction, as evidenced by the red/yellow island appearing in Figure 3A as opposed to the green plot obtained under backside irradiation, which leads to lower IQE values (Figure 3B). Notably, the largest IQE enhancement under frontside irradiation occurs below 450 nm, where WO_3_ absorbs light, and for BiVO_4_ layers thicker than 50 nm, which absorb a substantial fraction of incident light. The IQE in this irradiation mode maintains above 30% up to 450 nm for the best-performing electrodes, while it is seldom above 25% under backside irradiation, see for example the IQE traces for the WO_3_/BiVO_4_ electrode with 75 nm thick BiVO_4_ in Figure 3C.

At the same time, the IQE curves are similar under the two irradiation modes in the 450–520 nm range because only BiVO_4_ absorbs light at these wavelengths and process Ⓐ (Figure 1A) is predominantly active. Thus, these wavelength-dependent PEC analyses indicate that the performance of the WO_3_/BiVO_4_ photoanodes benefits from avoiding WO_3_ excitation. This condition occurs by selectively exciting BiVO_4_ under frontside irradiation, i.e., by shielding WO_3_ with BiVO_4_, and in both irradiation modes under excitation at wavelengths above the WO_3_ absorption onset (Figure 3C).

We then investigated the effect of WO_3_ shielding on the lifetime of photogenerated holes in the BiVO_4_ layer of the WO_3_/BiVO_4_ system. Previous work ascribed the transient absorption Δ*A* signal at 470 nm to trapped holes in BiVO_4_, based on experiments in the presence of hole scavengers.^18,21,31^ TAS proved an essential tool for studying charge carrier dynamics and diffusion in photocatalysis and photovoltaics.^32−35^ Therefore, TAS with detection at 470 nm was here employed to investigate the dynamics of photogenerated holes in both pure BiVO_4_ and WO_3_/BiVO_4_ electrode series upon backside and frontside excitation at 387 nm.

The Δ*A* signals recorded with pure BiVO_4_ electrodes were analyzed first. For this system, similar transient dynamics were obtained in the two irradiation modes. Figure S12 reports representative transient absorption spectra, while Figure S13 shows the transient decay Δ*A* profiles at 470 nm, which were fitted according to a biexponential decay model (eq 1).
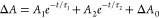
1In this equation, τ_1_ and τ_2_ are the lifetimes of the faster and slower decay processes typical of BiVO_4_, respectively, *A*_1_ and *A*_2_ are the weighted coefficients that represent the contribution of each of the two processes to the overall decay and Δ*A*_0_ is the offset (set at zero in the fitting).^21^ The fitting parameters for the BiVO_4_ electrodes (Table S1) are in line with literature reports on pure BiVO_4_. Regardless of the BiVO_4_ thickness , *A*_1_ and *A*_2_ account of ∼30 and 70% of the hole decay, respectively. The fast decay lifetime, which is associated with the recombination of trapped holes in BiVO_4_ with photopromoted free electrons, is independent of the BiVO_4_ layer thickness (τ_1_, ∼20 ps), because all electrodes are excited at the same pump wavelength (i.e., with the same energy excess with respect to the BiVO_4_ CB).^21,29^ On the other hand, τ_2_, which is ascribed to the recombination of trapped holes with trapped electrons, increases from ∼1 to 6.5 ns with increasing BiVO_4_ layer thickness as more holes get trapped in bulk sites.^28,36,37^

The decay signal of photoproduced holes in the BiVO_4_ layer of the WO_3_/BiVO_4_ electrodes series recorded at 470 nm under backside and frontside irradiation are reported in Figure 4 and Figures S14 and S15. Under frontside excitation, the Δ*A* signals decay slower than under backside excitation (Figure 4A–C). Indeed, under backside irradiation mode a significant fraction of 387 nm photons is absorbed by WO_3,_ leading to photoexcitation of electrons into its CB (the individual WO_3_ layer absorbs ca. 16% of 387 photons, Figure 1C). Therefore, many photoproduced charge carriers recombine through process Ⓑ (Figure 1A). This additional recombination channel leads to the abrupt Δ*A* drop observed during the first 400 ps following backside photoexcitation (Figure 4A). Furthermore, under frontside irradiation the Δ*A* signal recorded with the WO_3_/BiVO_4_ heterojunctions becomes progressively slower and comparable with those recorded with pure BiVO_4_ (Figure 4D–F and Figure S13).

**Figure 4:**
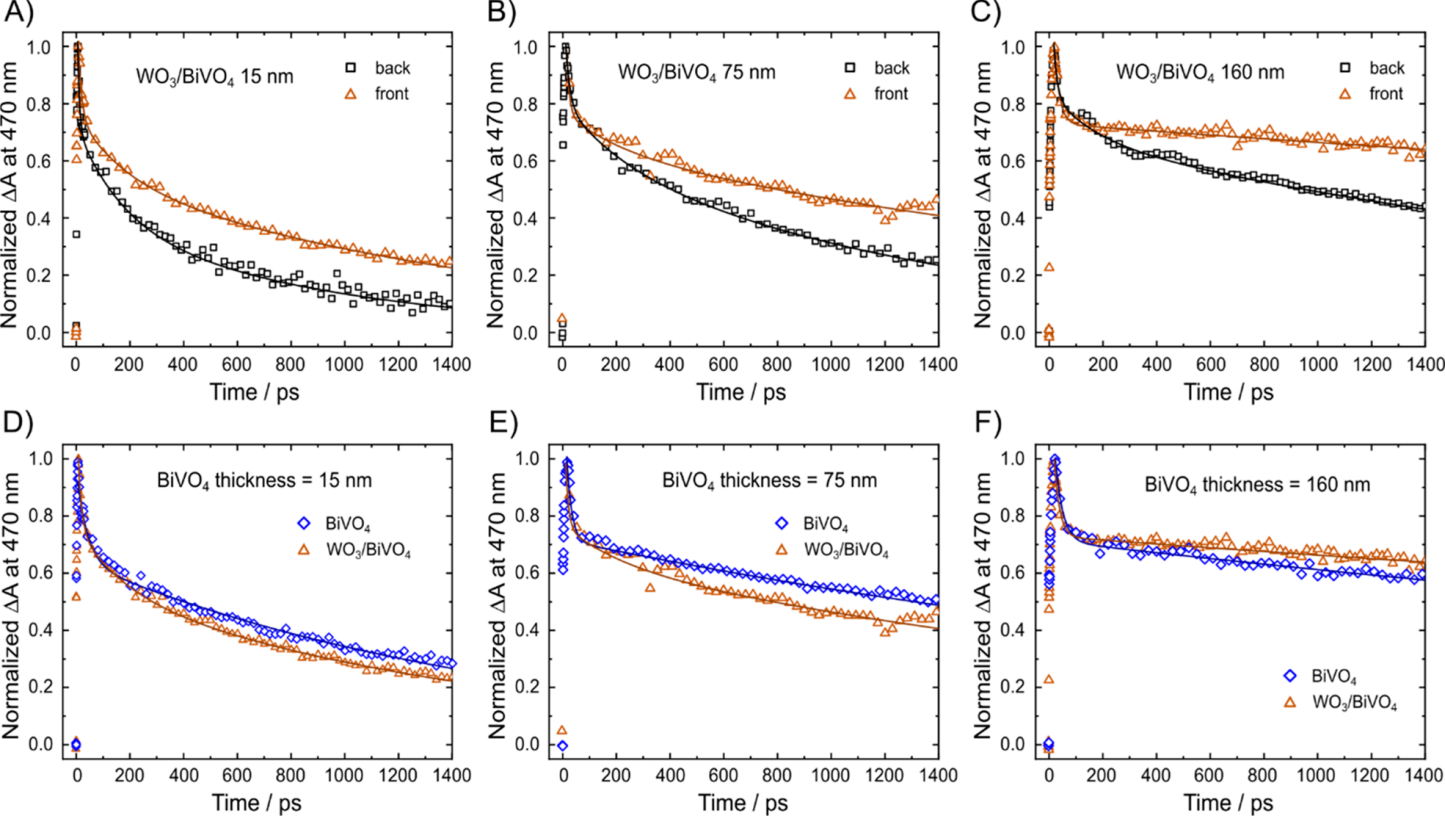
Comparison between the normalized TAS decay signals in (A–C) WO_3_/BiVO_4_ electrodes with different BiVO_4_ thicknesses recorded under backside (through WO_3_, black squares) or frontside (through BiVO_4_, red triangles) irradiation and in (D–F) WO_3_/BiVO_4_ (red triangles) and pure BiVO_4_ films (blue diamonds) with the same BiVO_4_ thickness recorded under frontside irradiation. The solid lines are the fitting curves according to eq 1 (BiVO_4_ films) or eq 2 (WO_3_/BiVO_4_ films). Excitation pump at 387 nm, TAS signal monitored at 470 nm.

In previous studies on the WO_3_/BiVO_4_ heterojunction, we evaluated the contribution of process Ⓑ to the overall Δ*A* decay signal by fitting the Δ*A* decay traces including an additional decay component in eq 1, to take into account also process Ⓑ.^26^ Here, we used the same approach to assess its contribution to the charge carrier dynamics in the two irradiation modes and fitted the Δ*A* decay with eq 2.
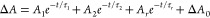
2where *τ*_r_ accounts for the additional recombination process and *A*_r_ is its weighted contribution.

We first fitted the dynamics recorded in the WO_3_/BiVO_4_ electrodes under backside excitation. The fitting parameters are reported in Table 1. In this configuration, the WO_3_ layer is irradiated directly and absorbs the same amount of light in all electrodes. Therefore, we expect that process Ⓑ has a comparable effect on the charge carrier dynamics at each BiVO_4_ layer thickness. Indeed, this process accounts for ca. 23 ± 8% of the holes decay, with a time constant *τ*_r_ of ∼200 ps (Table 1).

**Table 1:** Fitting Parameters of the TAS Dataset Collected with the WO_3_/BiVO_4_ Electrodes under Backside Excitation at 387 nm^a^

BiVO_4_ thickness (nm)	*A*_1_ (%)	τ_1_ (ps)	*A*_r_ (%)	τ_r_ (ps)	*A_2_ (%)*	τ_2_ (ns)
15	30 ± 1	7.4 ± 0.8	32 ± 3	170 ± 22	38 ± 3	0.98 ± 0.07
30	26 ± 2	6.2 ± 1.1	34 ± 3	189 ± 27	40 ± 3	1.9 ± 0.2
50	24 ± 2	7.8 ± 1.2	19 ± 2	156 ± 30	57 ± 2	1.99 ± 0.11
75	22 ± 1	15.8 ± 1.8	17 ± 2	215 ± 48	61 ± 3	1.44 ± 0.07
115	23 ± 2	22 ± 3	19 ± 2	210 ± 25	58.0 ± 0.3	3.57 ± 0.11
160	18 ± 1	13.1 ± 1.7	15 ± 1	159 ± 26	67.4 ± 0.9	3.05 ± 0.11

a Data fitted according to eq 2.

Under frontside irradiation, the BiVO_4_ layer in the heterojunction electrodes shields WO_3_ from light absorption as the BiVO_4_ layer thickness increases. Indeed, the percent amount of incident 387 nm photons absorbed by the WO_3_ underlayer in the coupled system progressively decreases (Figure 1C). Therefore, we sought to quantify the shielding effect of the BiVO_4_ layer in decreasing the extent of process Ⓑ in the WO_3_/BiVO_4_ electrodes. By assuming that process Ⓑ operates with its intrinsic time constant *τ*_r_ regardless of the excitation mode, we fitted the decay dynamics recorded under frontside irradiation by employing the *τ*_r_ previously extracted from the TAS signals recorded upon excitation in backside mode (Table 1). Because of the reduced amount of charge carriers generated in WO_3_, the weight of process Ⓑ in terms of the *A*_r_ parameter (Table 2) decreases with increasing the BiVO_4_ layer thickness. Additionally, as fewer charge carriers undergo process Ⓑ, *A*_2_ increases, suggesting that a larger number of photogenerated charge carriers recombines through the slower process. A 160 nm thick BiVO_4_ layer almost entirely absorbs the pump (Figure 1C), preventing WO_3_ excitation. Due to the lack of photoexcited electrons in the CB of WO_3_, the electrons photopromoted in the BiVO_4_ CB can only recombine with trapped holes in BiVO_4_, or flow into WO_3_ CB via process Ⓐ, resulting in better charge carrier separation. Consequently, the holes photogenerated in the BiVO_4_ layer of the WO_3_/BiVO_4_ heterojunction live longer than those in the individual 160 nm thick BiVO_4_ electrode (Figure 4F). This condition is akin to selective BiVO_4_ excitation in WO_3_/BiVO_4_ at wavelengths beyond WO_3_ absorption edge, which we previously observed extending the hole lifetimes compared to individual BiVO_4_.^26,29^

**Table 2:** Fitting Parameters of the TAS Dataset Collected with the WO_3_/BiVO_4_ Electrodes under Frontside Excitation at 387 nm^a^

BiVO_4_ thickness (nm)	*A*_1_ (%)	τ_1_ (ps)	*A*_r_ (%)	τ_r_ (ps)	*A*_2_ (%)	τ_2_ (ns)
15	25 ± 1	13 ± 1	20 ± 1	170	55 ± 1	1.55 ± 0.03
30	23 ± 3	15 ± 2	27 ± 3	189	50 ± 3	2.8 ± 0.4
50	21 ± 3	19 ± 7	13 ± 5	156	66 ± 2	3.0 ± 0.3
75	23 ± 3	10 ± 3	12 ± 4	215	65 ± 2	3.1 ± 0.4
115	20 ± 1	24 ± 4	4 ± 2	210	76.3 ± 0.8	5.4 ± 0.3
160	27 ± 1	21 ± 2	-	-	73.1 ± 0.3	10.6 ± 1.2

a Data fitted according to eq 2 using the *τ*_r_ values reported in Table 1.

Thus, TAS and PEC experiments suggest that light absorption by the BiVO_4_ layer in WO_3_/BiVO_4_ electrodes selectively suppresses process Ⓑ and promotes process Ⓐ, which leads to an increase of trapped hole lifetime in BiVO_4_. However, despite the promise of long-living holes in the heterojunction electrode with a 160 nm thick BiVO_4_ layer, it performs poorly compared to the most active heterojunction with the 75 nm thick BiVO_4_ layer. This latter electrode possesses an optimal matching between (i) WO_3_ sensitization to the visible light, (ii) photogenerated charge separation at the heterojunction, and (iii) efficient charge extraction toward the external circuit and the electrolyte. Indeed, thinner BiVO_4_ layers limit the electrode performance due to the low visible light absorption, while thicker films may suffer from a greater charge recombination owing to hole accumulation in the BiVO_4_ bulk under *operando* conditions.

In conclusion, we identified a shielding strategy to suppress the internal charge recombination occurring in the WO_3_/BiVO_4_ heterojunction due to WO_3_ excitation. Optimized light absorption in BiVO_4_ layers considerably suppresses this recombination channel. The best performing electrode tested in this work shows a 40% increase in the PEC performance under frontside irradiation compared to backside irradiation. Furthermore, these findings suggest that methods to suppress undesired wavelength-dependent recombination processes and optimize charge transport and surface catalysis are required to design efficient photoelectrodes based on type-II heterojunctions.

## Supporting Information Available

Experimental details, absorption spectra, XRD and FESEM analyses, linear sweep voltammetry tests, IQE and IPCE plots, TAS decay profiles, and fitting parameters (PDF)
