# Supplementary material for: Enhanced Charge Carrier Separation in WO3/BiVO4 Photoanodes Achieved via Light Absorption in the BiVO4 Layer
Source: ACS Appl Energy Mater. 2022 Oct 17;5(11):13142–8. doi: 10.1021/acsaem.2c02597 (PMC9709765; doi:10.1021/acsaem.2c02597)
Supplement: Supplementary file 1 — ae2c02597_si_001.pdf [file ae2c02597_si_001.pdf]

# SUPPORTING INFORMATION

## Enhanced Charge Carrier Separation in $WO_3$/$BiVO_4$ Photoanodes Achieved via Light Absorption in the $BiVO_4$ Layer

*Ivan Grigioni,[#,*] Annalisa Polo,[#] Maria Vittoria Dozzi,[#] Kevin G. Stamplecoskie,[&] Danilo H. Jara,[§] Prashant V. Kamat,[¶] and Elena Selli[#,*]*

[#] *Dipartimento di Chimica, Università degli Studi di Milano, Via Golgi 19, 20133 Milano, Italy*

[&] *Department of Chemistry, Queen's University, Kingston, Ontario K7L 3N6, Canada*

[§] *Facultad de Ingeniería y Ciencias, Universidad Adolfo Ibáñez, Av. Padre Hurtado 750, Viña del Mar, Chile*

[¶] *Radiation Laboratory, University of Notre Dame, Notre Dame, Indiana 46556, USA*

* Corresponding authors, Email: ivan.grigioni@unimi.it; elena.selli@unimi.it

# Supporting Information


| | | |
|---|---|---|
| **1** | **Experimental Section** | **page S3** |
| 1.1 | Materials | page S3 |
| 1.2 | Photoelectrodes preparation | page S3 |
| 1.3 | Photoelectrodes characterization | page S4 |
| 1.4 | Photoelectrochemical (PEC) characterization | page S4 |
| 1.5 | Transient absorption spectroscopy (TAS) | page S5 |
| **2** | **Supporting experimental data** | **page S6** |
| 2.1 | Absorption spectra, XRD and FESEM analyses | page S6 |
| 2.2 | Linear sweep voltammetry tests | page S9 |
| 2.3 | IQE and IPCE plots | page S11 |
| 2.4 | TAS decay profiles and fitting parameters of the $BiVO_4$ electrodes | page S13 |
| 2.5 | TAS decay profiles of the $WO_3/BiVO_4$ electrodes | page S15 |
| | **References** | **page S16** |

# 1. Experimental Section

## 1.1. Materials

The following chemicals were employed: tungsten(VI) ethoxide 99.8% (5% w/v in ethanol ethyl cellulose, citric acid 99%, ammonium vanadium oxide, bismuth(III), nitrate pentahydrate 98%(Aldrich), nitric acid; anhydrous sodium sulfate (Fisher Scientific).

## 1.2. Photoelectrodes preparation

$WO_3$ was prepared as described in our previous work.[1] Briefly, 1.0 mL of tungsten ethoxide, 5 wt.% in ethanol, was added inside a glovebox to 42 mg of citric acid acting as stabilizer. Once citric acid was completely dissolved, benzyl alcohol (0.3 mL) and ethyl cellulose (40 mg) were added to the solution, which was stirred overnight at 70° C to attain complete ethyl cellulose dissolution. The so obtained paste (with a 0.085 M tungsten content) is stable for several weeks. 100 μL of the paste were deposited on a 2.5 x 2.5 $cm^2$ fluorine-doped tin oxide (FTO) glass electrode (Pilkington Glass, TEC-7, thickness 2 mm), by spin coating at 6000 rpm for 30 s. The final spinning rate was reached with a three acceleration step program, i.e. 500 rpm $s^{-1}$ up to 1000 rpm, then 1000 rpm $s^{-1}$ up to 3000 rpm and finally 3000 rpm $s^{-1}$ up to 6000 rpm.[2] Prior to deposition, the FTO glass was cleaned by 15 min-long sonication, first in an aqueous soap solution, then in ethanol and finally in water. After coating, the so obtained film was dried at 80 °C for 1 h and then annealed at 500 °C for 8 h.

The $BiVO_4$ electrodes were prepared according to a procedure similar to that reported elsewhere.[2] Typically, 0.002 mol of $Bi(NO_3)_3$ and $NH_4VO_3$ were added to 6 mL of $HNO_3$ 23.3% containing 0.004 mol of citric acid. The mixture was stirred overnight to allow the complete dissolution of all precursors. The $BiVO_4$-based photoanodes were prepared on clean FTO by spinning 70 μL of the solution at 8000 rpm for 30 s with an acceleration rate of 6000 rpm $s^{-1}$. The films were then dried at 80 °C for 1 h and annealed at 500 °C for 1 h. The optical density of the $BiVO_4$ layer was modulated by repeating the spin coating procedure and the thermal treatment up to eight times; then the photoelectrodes were annealed at 500 °C for 8 h.

The $WO_3/BiVO_4$ combined electrodes were prepared by coating $WO_3$ electrodes with the solution containing the $BiVO_4$ precursors. Then the composite films were dried for 1 h at 80 °C and annealed at 500 °C for 1 h. The process was repeated up to eight times and the final $WO_3/BiVO_4$ electrodes were annealed at 500 °C for 8 h.

### 1.3. Photoelectrodes characterization

UV-visible absorption spectra were recorded using a Jasco V650 spectrophotometer. Electron microscopy images were obtained using a FEI Magellan-400 field emission scanning electron microscope (FESEM). The crystalline phase of the materials was determined through XRD analysis using a Philips PW1820 with Cu Kα radiation (λ = 1.54056 Å) at 40 mA and 40 kV.[3]

### 1.4. Photoelectrochemical (PEC) characterization

PEC measurements were carried out using a three electrode cell with an Ag/AgCl (3.0 M NaCl) reference electrode, a platinum gauze as a counter electrode and a PGSTAT204 (Autolab) potentiostat. The photoanodes were tested under backside and frontside illumination (through the $FTO/BiVO_4$ or $FTO/WO_3/BiVO_4$ interface). The light source was an Oriel, Model 81172 solar simulator providing AM 1.5G simulated solar illumination with 100 mW $cm^{-2}$ intensity (1 sun). 0.5 M $Na_2SO_4$ aqueous solution at pH 7 was used in electrochemical measurements. Linear sweep voltammetry experiments were carried out with a scan speed of 10 mV $s^{-1}$. The potential vs. Ag/AgCl was converted into the SHE scale using the following equation: $E_{SHE} = E_{AgCl} + 0.059\ pH + E^o_{AgCl}$, with $E^o_{AgCl}$ (3.0 M NaCl) = 0.210 V at 25°C.

Incident photon to current efficiency (IPCE) measurements were carried out using a 300 W Lot-Oriel Xe lamp equipped with a Lot-Oriel Omni-λ 150 monochromator and a Thorlabs SC10 automatic shutter. A 1.23 V bias vs. SHE was applied and the current was measured with a 10 nm step, within the 350 to 600 nm wavelength range. The incident light power was measured at each wavelength using a calibrated Thorlabs S130VC photodiode connected to a Thorlabs PM200 power meter. The IPCE was calculated at each wavelength using the following equation:

$$IPCE = \frac{1240 \times J}{P_\lambda \times \lambda} \times 100$$

where $J$ is the photocurrent density (mA cm$^{-2}$) and $P_\lambda$ (mW cm$^{-2}$) is the power of the monochromatic light at wavelength λ (nm). The internal quantum efficiency (IQE) was calculated by combining the IPCE spectrum with the absorption (A) spectrum of the photoanodes

$$IQE = \frac{IPCE}{1 - 10^{-A}}$$

### 1.5. Transient absorption spectroscopy (TAS)

Transient absorption spectroscopy (TAS) experiments with 387 nm laser pulse excitation were performed at the Radiation Laboratory, Notre Dame University, using a system based on a Ti:sapphire laser source (Clark MXR CPA-2010) generating pulses centered at 775 nm with a FWHM of 130 fs and 1 kHz repetition rate; 95% of the fundamental laser pulse was frequency doubled to λ = 387 nm and used as pump, while the remaining 5% was focused on a $CaF_2$ crystal to generate the white light continuum (WLC) probe. The transient spectra were recorded using a Helios transient absorption system of Ultrafast Systems. The pump energy fluence was 60 μJ cm$^{-2}$ during the experiments with excitation at 387 nm.

# 2. Supporting experimental data

## 2.1. Absorption spectra, XRD and FESEM analyses

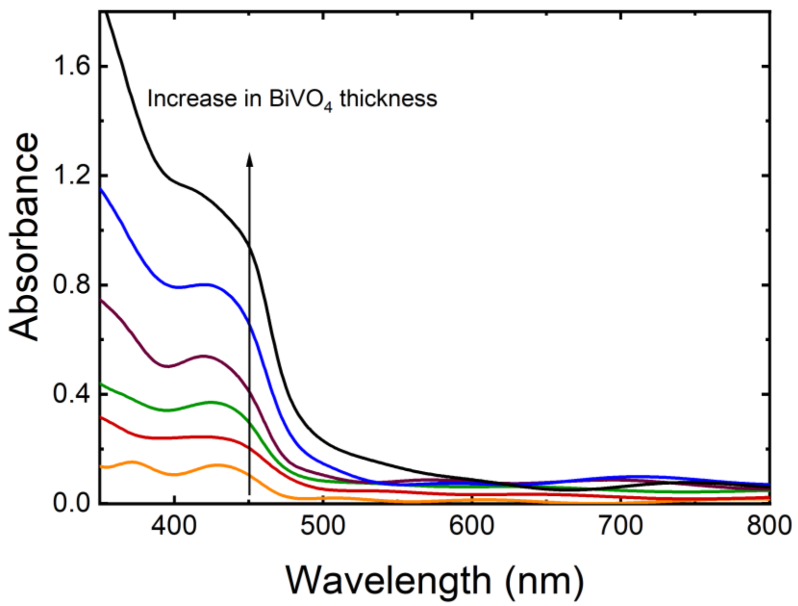


**Figure S1.** Absorption spectra of pure $BiVO_4$ electrodes.

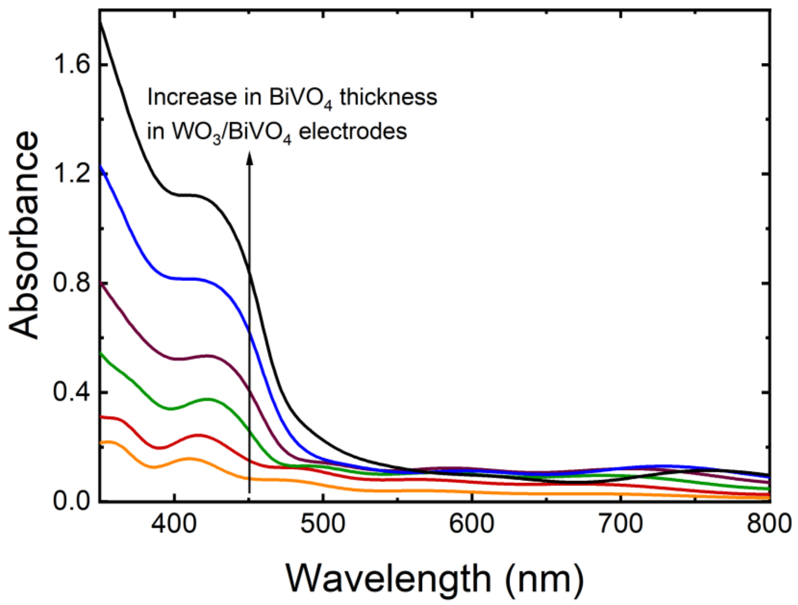


**Figure S2.** Absorption spectra of $WO_3/BiVO_4$ heterojunction electrodes.

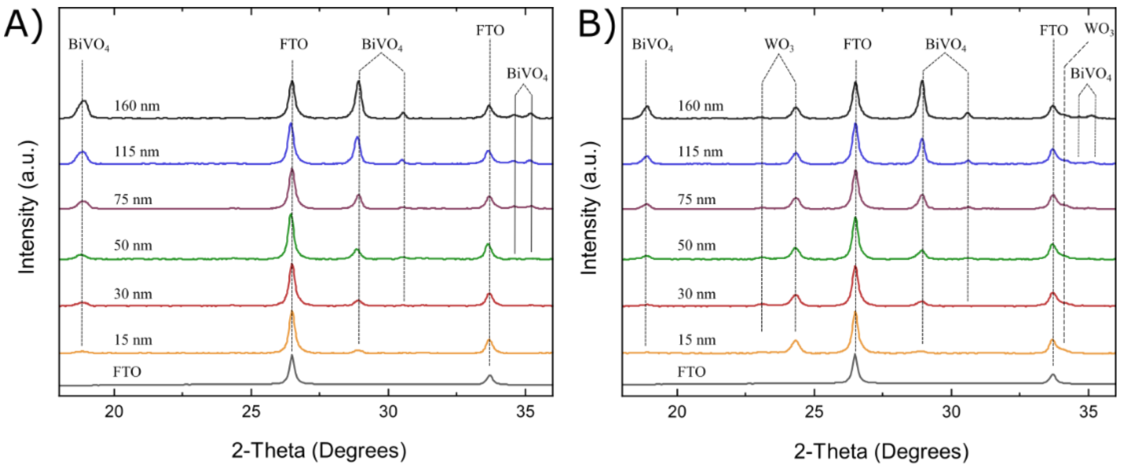


**Figure S3.** XRD diffractograms of A) the $BiVO_4$ and B) the $WO_3/BiVO_4$ series, with different $BiVO_4$ layer thickness (15 – 160 nm). The specific patterns of the fluorine doped tin oxide (FTO), scheelite $WO_3$, and monoclinic scheelite $BiVO_4$ are indicated.

Diffractograms of individual $BiVO_4$ films (Figure S3A) show the progressive intensity growth of the reflections ascribed to scheelite monoclinic $BiVO_4$, with increasing $BiVO_4$ thickness, while the XRD diffractograms of the heterojunction electrodes (Figure S3B) show constant intensity $WO_3$ peaks for different $BiVO_4$ thicknesses. Peaks of fluorine-doped tin oxide (FTO) appear in the two diffractograms series.

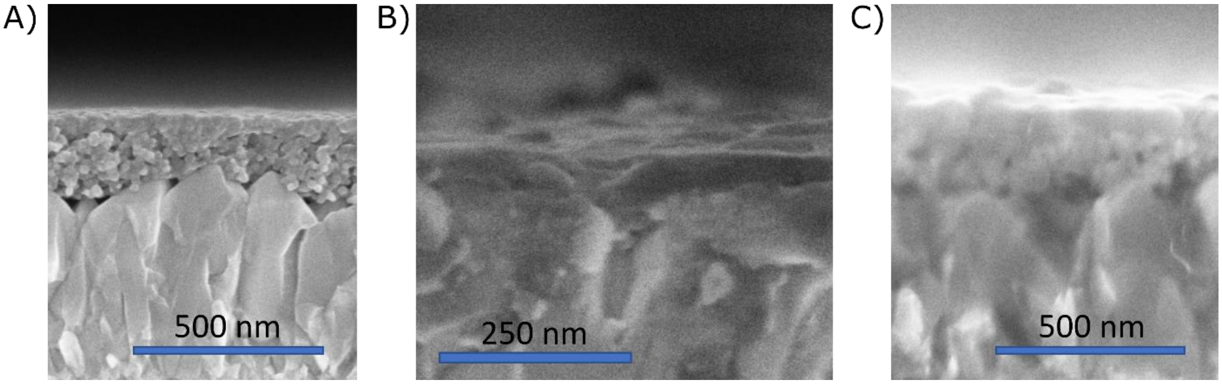


**Figure S4.** FESEM side-view images of A) the $WO_3$ photoanode and of the B) $BiVO_4$ and C) $WO_3/BiVO_4$ photoanodes with a 75 nm thick $BiVO_4$ layer obtained by 4 successive depositions.

The cross-section images of $WO_3$, $BiVO_4$ and $WO_3/BiVO_4$ electrodes with a 75 nm thick $BiVO_4$ layer (obtained upon 4 $BiVO_4$ depositions) are reported in Figure S4. In previous work from our group (ref. 3), we carried out cross-sectional FESEM analyses on $WO_3/BiVO_4$ films with different $BiVO_4$ thicknesses and calculated the $BiVO_4$ extinction coefficient at 420 nm, which has been here employed to estimate the thickness of the $BIVO_4$ layer in the two photoanodes series.

## 2.2. Linear sweep voltammetry tests

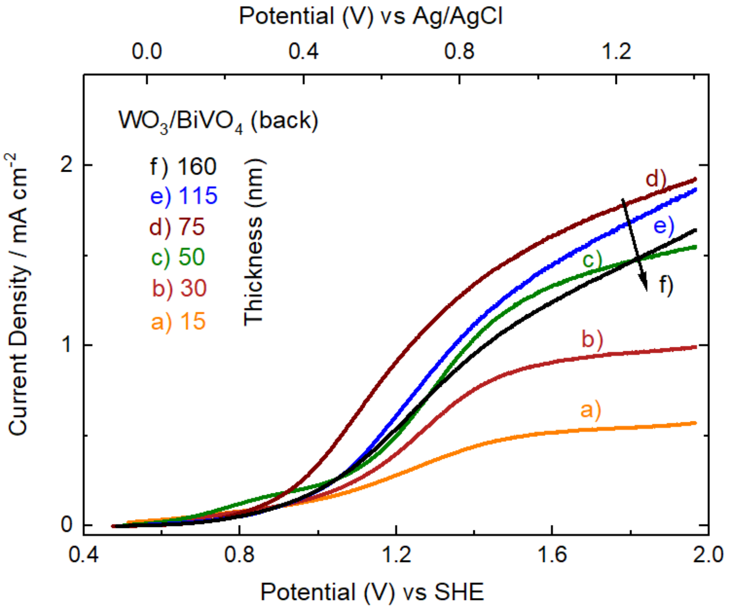


**Figure S5.** Linear sweep voltammetry curves of the $WO_3$/$BiVO_4$ heterojunction electrodes with different $BiVO_4$ layer thickness, recorded in contact with a 0.5 M $Na_2SO_4$ solution under backside irradiation.

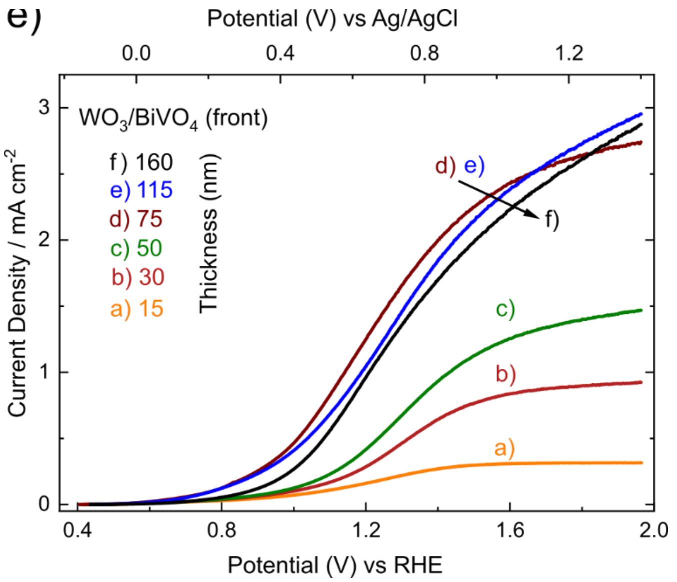


**Figure S6.** Linear sweep voltammetry curves of the $WO_3$/$BiVO_4$ heterojunction electrodes with different $BiVO_4$ layer thickness, recorded in contact with a 0.5 M $Na_2SO_4$ solution under frontside irradiation.

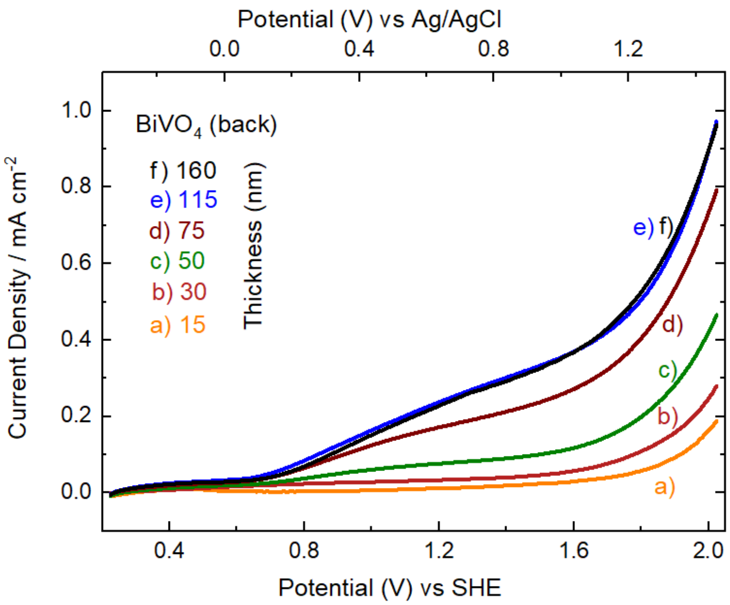


**Figure S7.** Linear sweep voltammetry curves of the pure $BiVO_4$ electrodes with different $BiVO_4$ layer thickness, recorded in contact with a 0.5 M $Na_2SO_4$ solution under backside irradiation.

## 2.3. IQE and IPCE plots

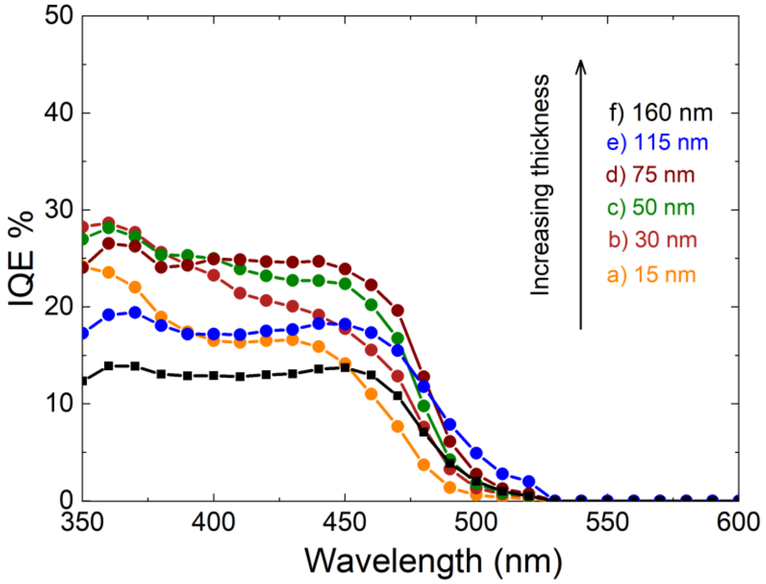


**Figure S8.** Internal quantum efficiency (IQE) of the $WO_3/BiVO_4$ heterojunction electrodes with different $BiVO_4$ layer thickness, measured in contact with a 0.5 M $Na_2SO_4$ solution at 1.23 $V_{SHE}$ under backside irradiation.

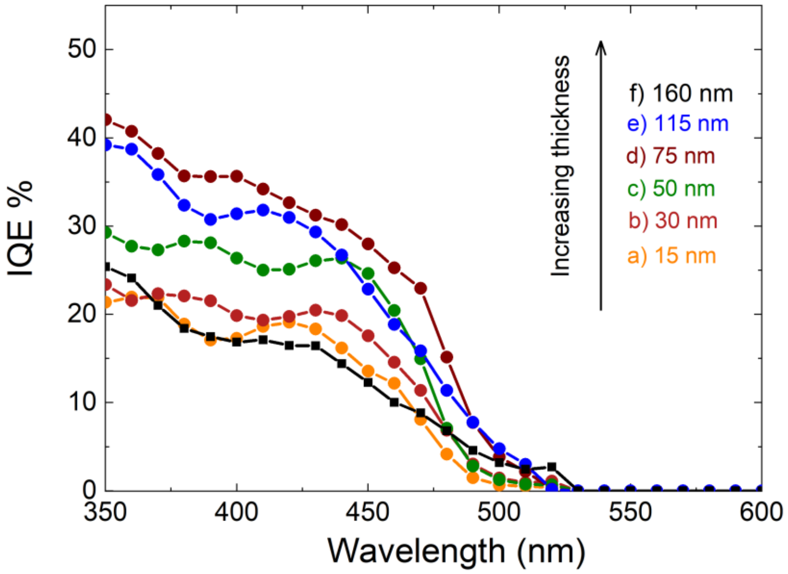


**Figure S9.** Internal quantum efficiency (IQE) of the $WO_3/BiVO_4$ heterojunction electrodes with different $BiVO_4$ layer thickness, measured in contact with a 0.5 M $Na_2SO_4$ solution at 1.23 $V_{SHE}$ under frontside irradiation.

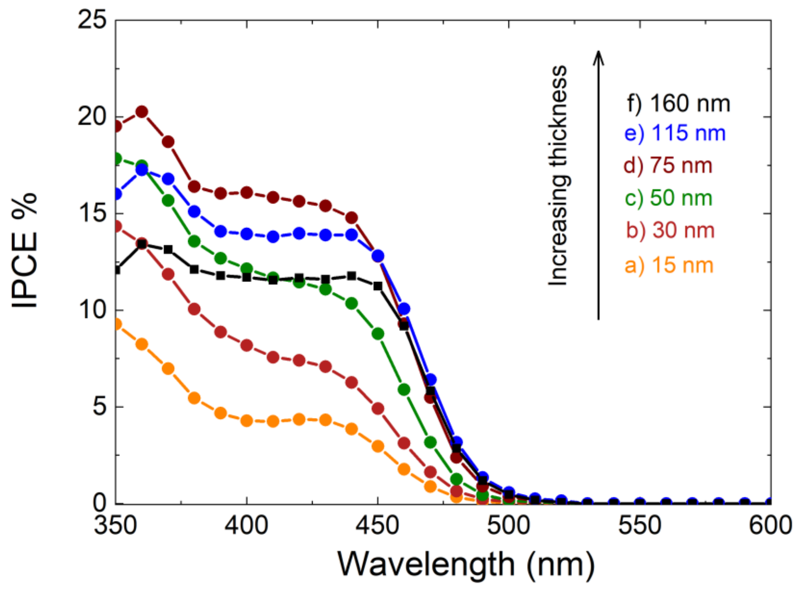


**Figure S10.** Incident photon to current efficiency (IPCE) of the $WO_3/BiVO_4$ heterojunction electrodes with different $BiVO_4$ layer thickness, measured in contact with a 0.5 M $Na_2SO_4$ solution at 1.23 $V_{SHE}$ under backside irradiation.

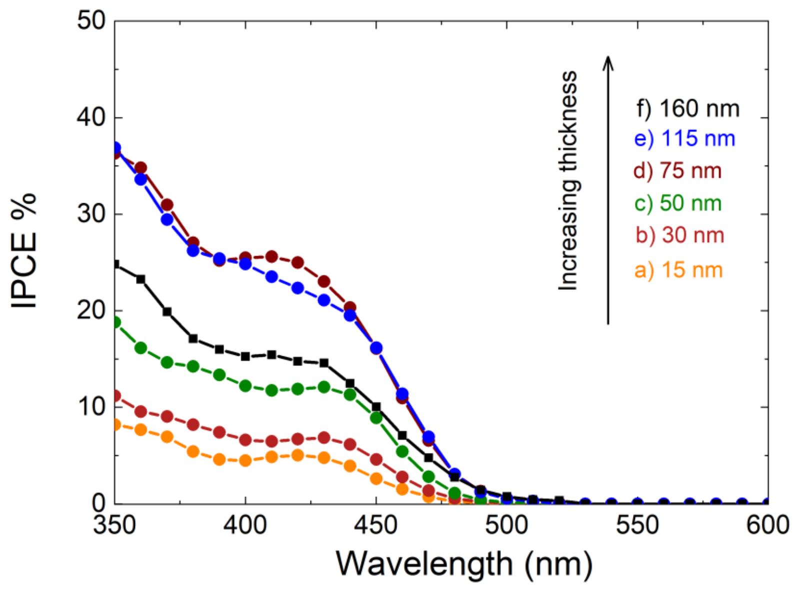


**Figure S11.** Incident photon to current efficiency (IPCE) of the $WO_3/BiVO_4$ heterojunction electrodes with different $BiVO_4$ layer thickness, measured in contact with a 0.5 M $Na_2SO_4$ solution at 1.23 $V_{SHE}$ under frontside irradiation.

## 2.4. TAS decay profiles and fitting parameters of the $BiVO_4$ electrodes

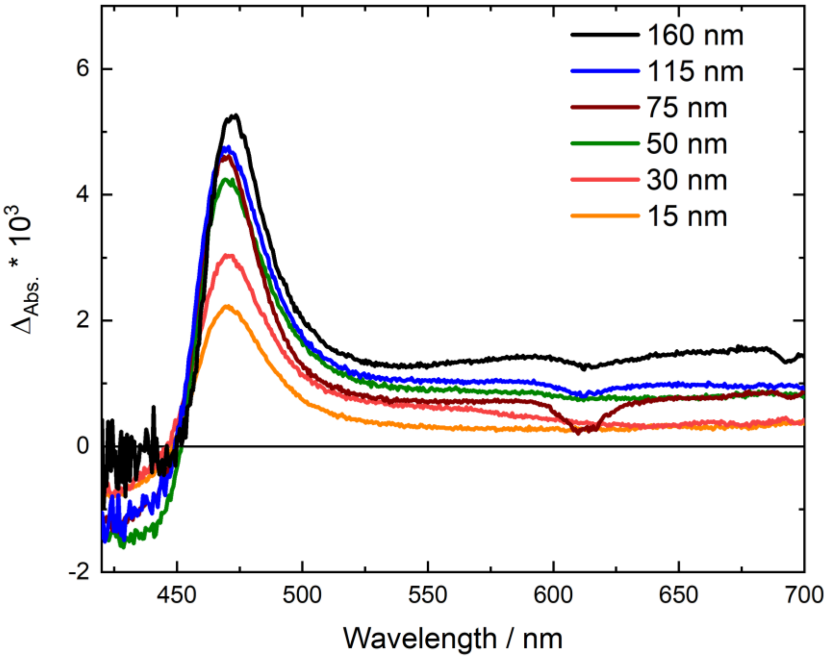


**Figure S12.** Transient absorption spectra of pure $BiVO_4$ electrodes with different $BiVO_4$ layer thickness (15 – 160 nm) recorded 10 ps after excitation at 387 nm.

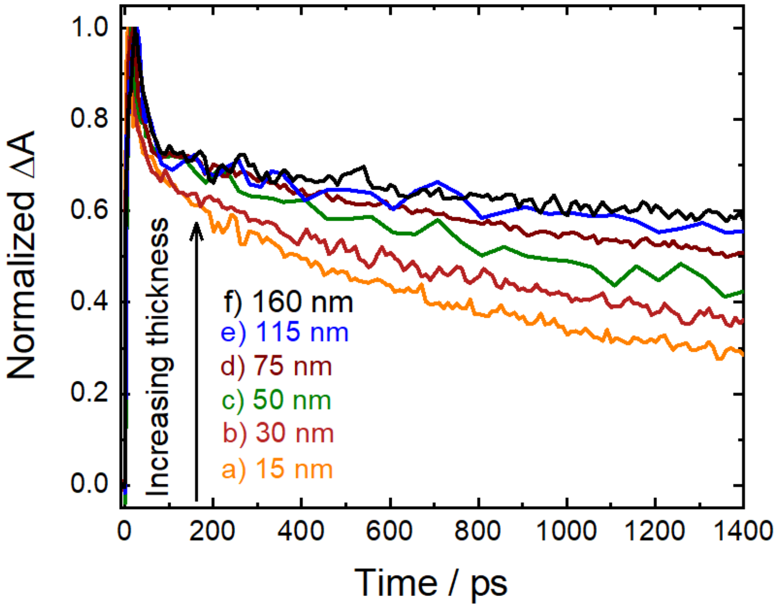


**Figure S13.** Transient absorption profiles at 470 nm in pure $BiVO_4$ electrodes with different $BiVO_4$ layer thickness (15 – 160 nm) upon excitation at 387 nm.

**Table S1.** Fitting parameters, according to Equation 1, of the $\Delta A$ transient decay signal at 470 nm shown in Figure S11.

| $BiVO_4$ thickness / nm | $A_1$ % | $\tau_1$ / ps | $A_2$ % | $\tau_2$ / ns |
|---|---|---|---|---|
| 15 | 35.9 ± 0.9 | 28.0 ± 1.7 | 64.1 ± 0.5 | 1.27 ± 0.02 |
| 30 | 34.6 ± 0.8 | 18.2 ± 0.9 | 65.4 ± 0.3 | 2.12 ± 0.03 |
| 50 | 28.3 ± 1.1 | 20.0 ± 1.9 | 71.7 ± 0.6 | 2.59 ± 0.09 |
| 75 | 28.9 ± 0.5 | 18.2 ± 0.9 | 71.1 ± 0.2 | 3.68 ± 0.05 |
| 115 | 29.7 ± 1.7 | 19.5 ± 2.2 | 70.3 ± 0.7 | 5.5 ± 0.4 |
| 160 | 28.8 ± 0.6 | 27.8 ± 1.8 | 71.2 ± 0.3 | 6.5 ± 0.2 |

## 2.5. TAS decay profiles of the $WO_3/BiVO_4$ electrodes

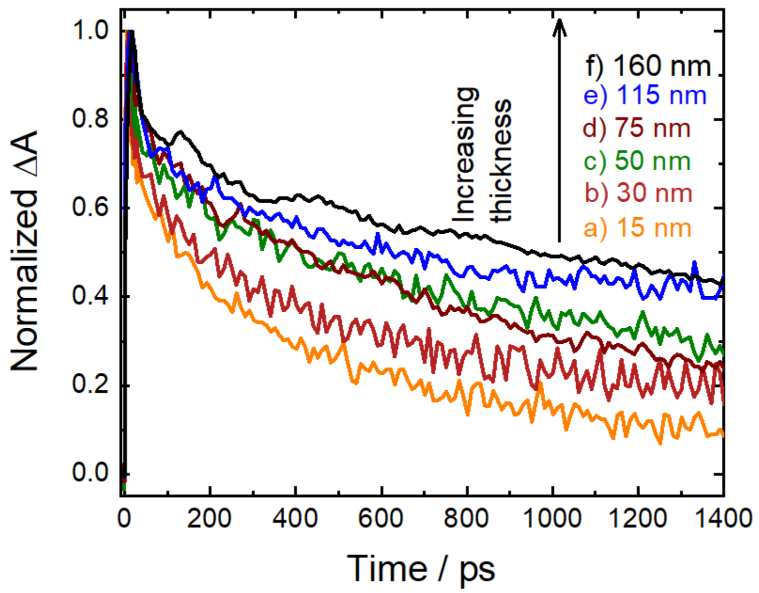


**Figure S14.** Transient absorption profiles at 470 nm of the $WO_3/BiVO_4$ electrodes with different $BiVO_4$ layer thickness (15 – 160 nm) recorded upon backside excitation at 387 nm.

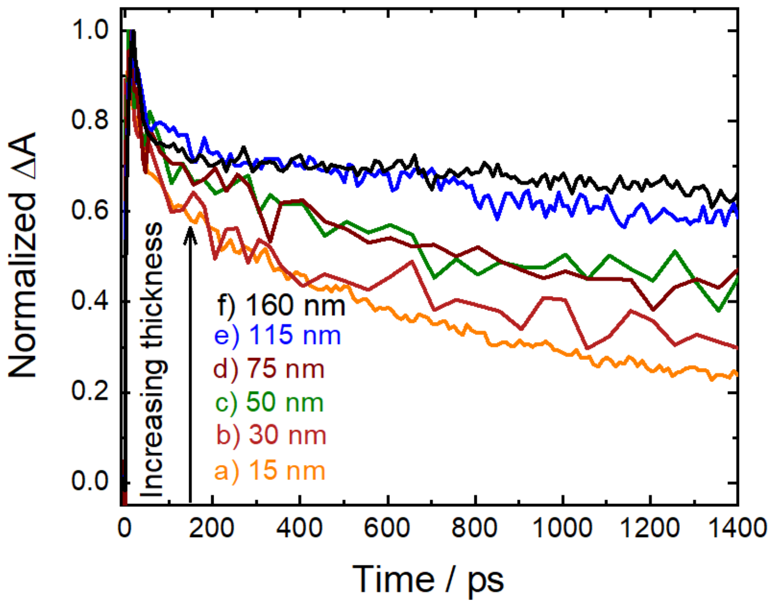


**Figure S15.** Transient absorption profiles at 470 nm of the $WO_3/BiVO_4$ electrodes with different $BiVO_4$ layer thickness (15 – 160 nm) recorded upon frontside excitation at 387 nm.

## References

(1) Grigioni, I.; Stamplecoskie, K. G.; Selli, E.; Kamat, P. V. Dynamics of Photogenerated Charge Carriers in $WO_3/BiVO_4$ Heterojunction Photoanodes. *J. Phys. Chem. C* **2015**, *119*, 20792–20800. https://doi.org/10.1021/acs.jpcc.5b05128.

(2) Grigioni, I.; Stamplecoskie, K. G.; Jara, D. H.; Dozzi, M. V.; Oriana, A.; Cerullo, G.; Kamat, P. V.; Selli, E. Wavelength-Dependent Ultrafast Charge Carrier Separation in the $WO_3/BiVO_4$ Coupled System. *ACS Energy Lett.* **2017**, *2*, 1362–1367. https://doi.org/10.1021/acsenergylett.7b00216.

(3) Grigioni, I.; Di Liberto, G.; Dozzi, M. V.; Tosoni, S.; Pacchioni, G.; Selli, E. $WO_3/BiVO_4$ Photoanodes: Facets Matching at the Heterojunction and $BiVO_4$ Layer Thickness Effects. *ACS Appl. Energy Mater.* **2021**, *4*, 8421–8431. https://doi.org/10.1021/acsaem.1c01623.
